# The maize *INDETERMINATE1 *flowering time regulator defines a highly conserved zinc finger protein family in higher plants

**DOI:** 10.1186/1471-2164-7-158

**Published:** 2006-06-19

**Authors:** Joseph Colasanti, Reynald Tremblay, Ada YM Wong, Viktoriya Coneva, Akiko Kozaki, Barbara K Mable

**Affiliations:** 1 Department of Molecular and Cellular Biology, University of Guelph, Guelph, Ontario, N1G 2W1, Canada; 2 Department of Biology, Shizuoka University, Ohya 836, Shizuoka, Japan; 3 Division of Environmental and Evolutionary Biology, University of Glasgow, Glasgow G12 8QQ, Scotland, UK

## Abstract

**Background:**

The maize *INDETERMINATE1 *gene, *ID1*, is a key regulator of the transition to flowering and the founding member of a transcription factor gene family that encodes a protein with a distinct arrangement of zinc finger motifs. The zinc fingers and surrounding sequence make up the signature ID domain (IDD), which appears to be found in all higher plant genomes. The presence of zinc finger domains and previous biochemical studies showing that ID1 binds to DNA suggests that members of this gene family are involved in transcriptional regulation.

**Results:**

Comparison of *IDD *genes identified in Arabidopsis and rice genomes, and all *IDD *genes discovered in maize EST and genomic databases, suggest that *ID1 *is a unique member of this gene family. High levels of sequence similarity amongst all *IDD *genes from maize, rice and Arabidopsis suggest that they are derived from a common ancestor. Several unique features of *ID1 *suggest that it is a divergent member of the maize *IDD *family. Although no clear *ID1 *ortholog was identified in the Arabidopsis genome, highly similar genes that encode proteins with identity extending beyond the ID domain were isolated from rice and sorghum. Phylogenetic comparisons show that these putative orthologs, along with maize *ID1*, form a group separate from other *IDD *genes. In contrast to *ID1 *mRNA, which is detected exclusively in immature leaves, several maize *IDD *genes showed a broad range of expression in various tissues. Further, Western analysis with an antibody that cross-reacts with ID1 protein and potential orthologs from rice and sorghum shows that all three proteins are detected in immature leaves only.

**Conclusion:**

Comparative genomic analysis shows that the *IDD *zinc finger family is highly conserved among both monocots and dicots. The leaf-specific *ID1 *expression pattern distinguishes it from other maize *IDD *genes examined. A similar leaf-specific localization pattern was observed for the putative ID1 protein orthologs from rice and sorghum. These similarities between *ID1 *and closely related genes in other grasses point to possible similarities in function.

## Background

Comparison of gene families from different plants provides insights into gene functions that have evolved from common ancestral genes in order to meet unique requirements of each species. The availability of the complete genome sequences of two plants, the small flowering dicot, *Arabidopsis thaliana *[1], and the agronomically important monocot, rice (*Oryza sativa*), [2,3] allows for analysis of gene functions that are shared between these diverse species. An important but little understood function that is common to all higher plants involves the mechanism underlying the signals that control time to flowering. Recent studies suggest that some of the genes that regulate the floral transition in *Arabidopsis *have homologous counterparts with a similar function in rice [4,5]. Conversely, other genes that control flowering in rice and maize have no apparent functional equivalent in *Arabidopsis *[6,7]. The transition to flowering is mediated by a complex network of pathways that integrate both environmental and developmental signals [8] so it is not surprising that some gene functions are conserved, whereas other genes appear to have evolved distinctive functions to meet the specific floral transition requirements within a particular species.

The maize *INDETERMINATE1 *gene *, ID1*, may exemplify a situation where one member of a gene family has acquired a function that is distinct from other family members. Loss-of-function *id1 *mutants remain in a prolonged state of vegetative growth and form aberrant flowers [9,10]. So far, *ID1 *is the only gene known to have a major role in controlling the transition to flowering in maize. Analysis of genes related to *ID1 *could provide insights into how modification of an ancestral function has lead to its current function and could reveal whether functional homologs of *ID1 *exist in other plant species.

Recent studies in species such as Arabidopsis and rice show that transcription factor genes are key components of genetic networks that control the transition to flowering [11]. Presumably loss of key regulators would affect the expression of batteries of downstream genes that mediate the complex transition process [12]. Evidence for recent genome duplications throughout eukaryotic evolution [13-15], might imply that the functions of many genes are redundant and may not be uncovered by single-gene mutation analysis. Therefore it is of interest to analyze entire gene families at the genomic level in order to predict possible gene functions and to examine how new functions evolve from those of shared ancestral genes. This may be especially significant in the case of transcription factors, which are modular in nature; i.e., they typically contain a DNA-binding domain and a separate activator or repressor domain. The latter domain is important for controlling the expression of genes that contain *cis *elements that are recognized by the DNA-binding domain [16]. Diverse new functions may be generated from the "mixing and matching" of DNA binding domains with other elements of the proteins, resulting in the control of other networks of genes.

The ID1 protein contains Cys2His2 type zinc finger motifs that are related to animal regulator proteins such as *Krüppel *in *Drosophila *and *Xfin *in *Xenopus *[17]. Zinc fingers are one of the most abundant class of regulatory proteins represented in the eukaryotic genomes that have been sequenced to date [18,19] and, in Arabidopsis, zinc fingers apparently comprise the largest group of putative regulatory genes [20]. As the complete sequences of more genomes become available, greater effort is being directed at comparative studies of genes and gene families from different organisms [21]. Genome-based approaches focus on questions about specific genes and gene families with regard to the function of ancestral genes, and how these genes evolved to suit the specific developmental requirements of particular organisms.

Here we analyze the zinc finger proteins encoded by the family of *IDD *genes found in two plants with sequenced genomes, rice and Arabidopsis, and compare them with the *ID1 *gene and all known *ID1*-related genes identified in available maize sequence databases. Comparison of the sequences and structures of the *IDD *genes in these three species suggests that maize *ID1 *is a unique member of this gene family, although putative functional homologs of *ID1 *were discovered in the related grass species rice, as well as sorghum.

## Results

### Maize *ID1 *defines the ID-domain containing transcription factor gene family

**Figure 1:**
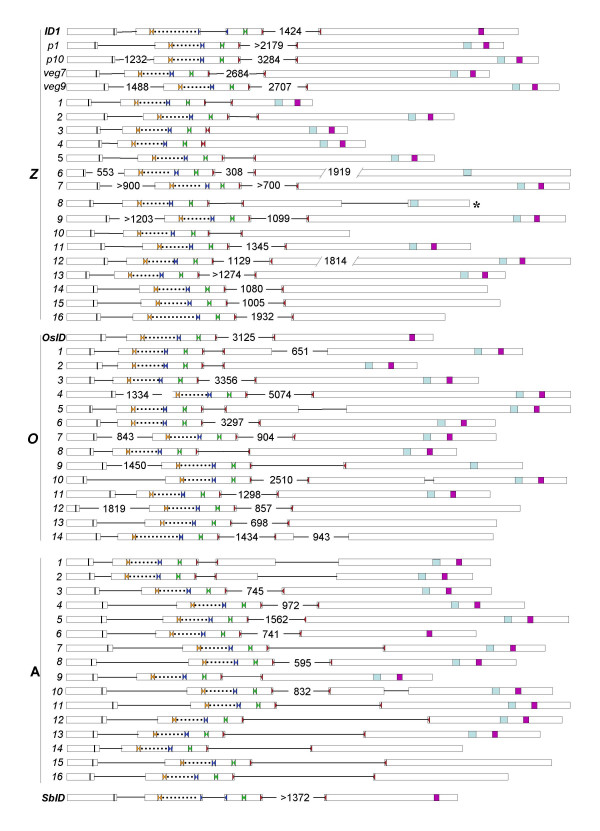
**Gene structure of *IDD *genes from maize, rice and Arabidopsis and *SbID *from sorghum**. Exons are represented by the open boxes and introns by black lines between boxes. The putative NLS is shown by a dark bar in the first exon; each zinc finger is shown by facing colored triangles (ZF1, orange; ZF2, blue; ZF3, green and ZF4, red). The amino acid spacer between ZF1 and ZF2 is represented by a dotted line. Numbers represent intron size; numbers between forward slashes signify exon size. Introns sizes preceded by '>' indicate that the full sequence is not known but that it is at least as long as indicated. The C-terminal conserved motifs, MSATALLQKAA (light blue box) and TR/LDFLG (purple box) are in the last exon. The asterisk (*) indicates that a stop codon could not be identified. *Z *= *ZmIDD*, *O *= *OsIDD*, *A *= *AtIDD*.

**Figure 2:**
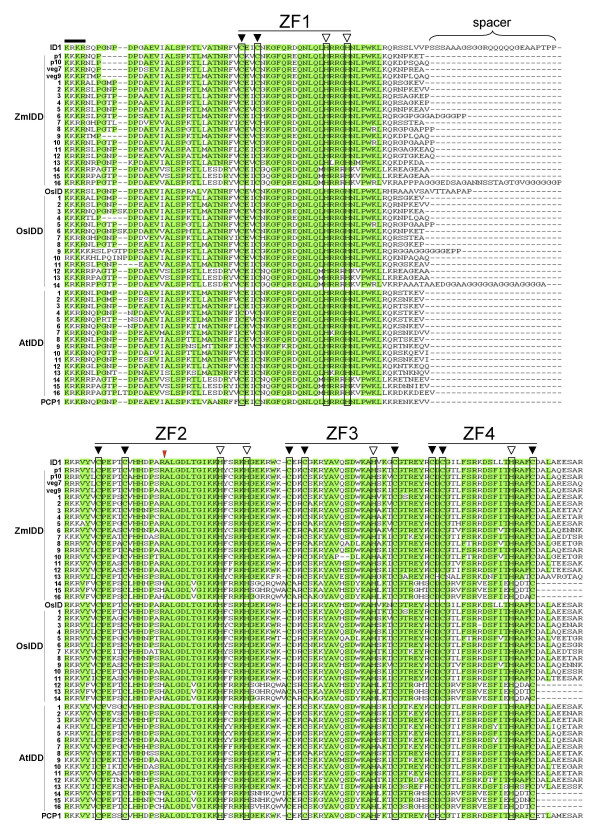
**Alignment of deduced ID-domain amino acid sequences of maize, rice and Arabidopsis *IDD *genes, as well as *PCP1 *from potato**. Alignment of the ID domain. Green shaded areas represent amino acid identity in 70% or more of the sequences. The position of each zinc finger is by a bar and cysteine (C) and histidine (H) residues boxed; filled triangle indicate conserved C residues and open triangle indicate conserved H residues. (For the sake of simplicity, we have renamed the zinc finger order (Z1, Z2, Z3 and Z4); in a previous study (Kozaki et al., 2004) the order was Z1, Z3, Z2 and Z4.) A thick bar at the N-terminal region of the ID-domain shows the putative NLS sequence. The pink triangle represents the position intron unique to *ID1 *and *SbID*.

The *ID1 *gene was tagged with a *Ds2 *transposable element that facilitated isolation of a full-length coding sequence [22]. In initial attempts to isolate the *ID1 *coding sequence, cDNA libraries made from 3-leaf seedling, vegetative apex, and developing inflorescence were screened with a portion of the *ID1 *genomic sequence. More than 50 hybridizing clones were isolated from these libraries and found to correspond to 4 different classes of cDNAs that were similar to *ID1*, however none of them corresponded to the maize *ID1 *gene. These clones were designated *ID*-domain genes (*IDD*), and given the names *ZmIDDp1*, *ZmIDDp10*, *ZmIDDveg7 *and *ZmIDDveg9*. Comparison of the *ID1 *cDNA sequence with the four related cDNA clones revealed that they shared a highly conserved region of ~170 amino acids (Figures 1, 2). This conserved ID-domain (IDD), has a putative nuclear localization sequence at the N-terminal border consisting of charged lysine (K) and arginine (R) residues [23], followed by four distinct zinc finger motifs. Two of the ID-domain zinc fingers have the hallmarks of the TFIIIA zinc finger class of proteins, whereas the other two zinc fingers have atypical structures and were identified by visual inspection of the deduced amino acid sequence and biochemical analysis of DNA binding properties [24].

**Figure 3:**
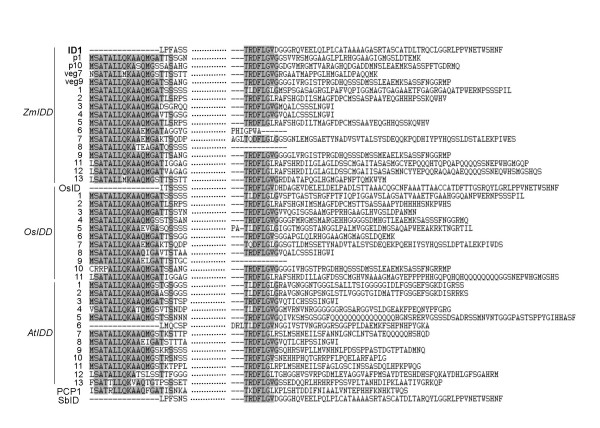
**Alignment of C-terminal amino acid sequences encoded by maize, rice and Arabidopsis *IDD *genes, *SbID *from sorghum and *PCP1 *from potato**. The C-terminal regions of IDD peptides that contain either the MSATALLQKAA or TR/LDFLG conserved domains, or both, shaded in gray. The dotted line represents the non-conserved amino acid sequence separating the two conserved motifs.

Low-stringency BLAST searches of available NCBI nucleotide and protein databases with *ID1 *and the maize *IDD *genes (*ZmIDD*) as query sequences revealed significant similarity to zinc finger proteins from animals and yeast, as well as plants. The highest level of similarity, however, was found in a particular sub-group of plant zinc finger genes, as described below. That is, the extent of similarity of maize *ID1 *and *ZmIDD *genes to animal and yeast sequences was limited mostly to the positioning of the zinc-interacting C and H residues and the conserved hydrophobic residues that define this protein class. [25], whereas the plant sequences have extensive similarity throughout the ID-domain (Figure 2) as well as some conserved domains in the C-terminal region (Figure 3). ID-domain proteins therefore define a sub-group of zinc fingers that is highly conserved in higher plants.

A complete, annotated maize genome sequence is not yet available, but a large number of sequences have been collected in the GSS (Genome Survey Sequence) database, which contains gene-enriched maize DNA sequences derived by methylation filtration [13,26], High-Cot enrichment and other methods that reduce repetitive sequences [44]. We extracted and reconstructed 20 partial and full-length maize *IDD *genes from the GSS collection. A large collection of maize EST and cDNA sequences recently made available by biotechnology companies and distributed by the National Corn Growers Association (NCGA) were used to complete most maize *IDD *sequences [[47]]. Derivation of *IDD *cDNAs from these resources allowed the intron-exon structure and full-length versions of 20 *IDD *genes to be deciphered, except for *ZmIDD8*, for which a definitive stop codon could not be assigned (Figure 1). Only one *IDD *gene, *ZmIDD7*, was found in the NCGA collection that was not found also in GSS or the public EST/cDNA databases. This suggests that the combined sequences of these two databases have extensive overlap and likely represent a large portion of the maize gene space. A similar level of coverage was reported for 78 full-length cDNAs that were used to determine the level of coverage of the GSS database and public EST and cDNA collections.

Although the Arabidopsis and rice genomes each have clearly identifiable *IDD *genes (described below, Figure 2), no phenotype associated with the loss of *IDD *gene function has been reported yet in these species. Maize has at least 20 *IDD *genes (Figure 2), but so far only mutations in the *ID1 *gene have been shown to have an effect on plant growth; i.e., a severe effect on flowering time and floral development [22].

### Maize *IDD *and *ID1 *genes have diverse expression patterns

**Figure 4:**
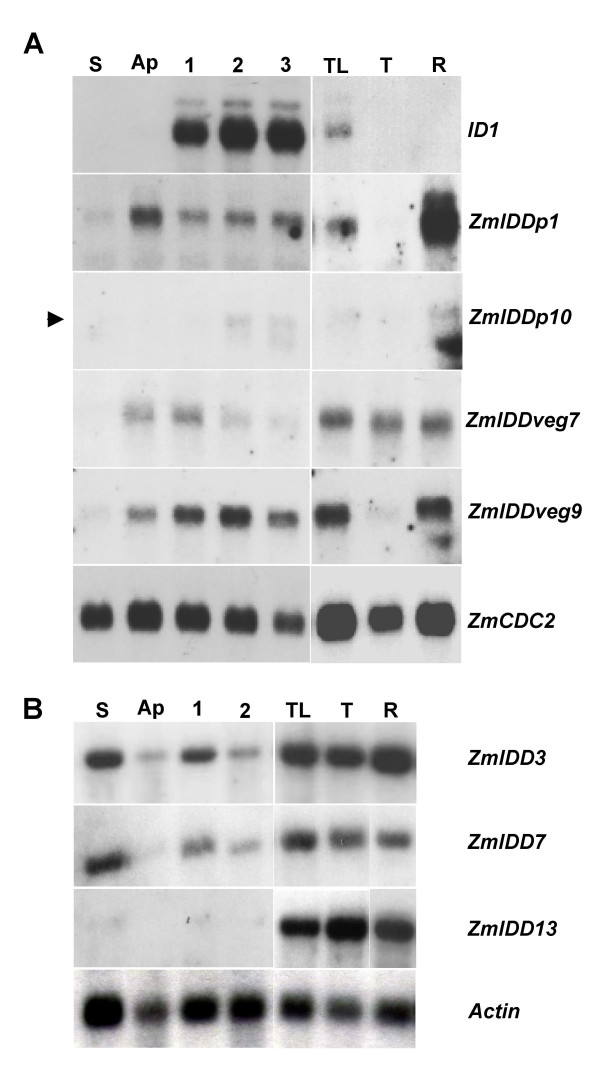
**Northern blot showing expression of 7 maize *IDD *genes compared to *ID1***. Gene-specific probes are indicated on the right side. Tissues used include: S, stem below the apical region, Ap, apical region of plant, including 2 cm above apical meristem and leaf primordia. Sections 1, 2 and 3 are immature leaf sections from 2–4 cm, 4–6 cm and 6–8 cm above the apex, respectively. TL, immature flag leaf surrounding tassel primordium; T, pre-anthesis tassel; R, roots. The same B73 plant with 7 visible leaves was used for sections S, Ap, 1, 2 and 3. (**A**) Gene-specific probes derived from cDNAs of *ZmIDDp1*, *ZmIDDp10*, *ZmIDDveg7 *and *ZmIDDveg9*. The *ZmCDC2 *probe, which is detected in tissues with actively dividing cells, was used as a control [38]. An arrowhead indicates the position of the *ZmIDDp10 *band. (**B**) Blot hybridized with specific probes to *ZmIDD3*, *ZmIDD7*, *ZmIDD13 *and an actin-specific probe.

A previous study found that *ID1 *mRNA is expressed only in immature leaves and is not detectable in the shoot apical meristem region, leaf primordia or in mature leaves [22]. This highly specific expression pattern suggests that *ID1 *has a role in mediating the production or the transmission of a leaf-derived floral inductive signal [10]. To compare the *ID1 *expression pattern with other maize *IDD *genes we used Northern hybridization and gene-specific probes to the 4 IDD genes isolated from maize cDNA libraries: *ZmIDDp1*, *ZmIDDp10*, *ZmIDDveg7 *and *ZmIDDveg9*, as well as 3 other *ZmIDD *genes, *ZmIDD3*, *ZmIDD7*, *ZmIDD13*, with probes isolated by PCR (Figure 4). We found that each of these 7 maize *IDD *genes had distinct expression patterns and that none of them exhibited tissue-specific expression similar to *ID1 *(Figure 4). In all cases the range of expression is broader than *ID1*, with mRNA of *ZmIDD *genes detected in most plant tissues (Figure 4). For example, whereas *ID1 *mRNA is confined to immature leaves and is clearly absent from other shoot tissues, *ZmIDDp1 *mRNA is detected in all vegetative tissues, and is especially abundant in tissues enriched for shoot and root apical meristems (Figure 4A). Similar to *ID1*, the *ZmIDDp10 *gene is detected in immature leaves, albeit at a much lower level, but it is also expressed at low levels in root and is barely detectable in floral tissues (Figure 4A). *ZmIDDveg9*, which was isolated from a vegetative shoot apex cDNA library, is detected in apical meristem-enriched tissues, but is also found in immature leaves, roots and, at a lesser level, in tassels (Figure 4A). In contrast, *ZmIDDveg7*, which was isolated from the same vegetative meristem cDNA library as *ZmIDDveg9*, shows a similar expression pattern, except that it is expressed at higher levels in floral tissues and it is detected in apical regions and leaf primordia but to a lesser extent in more developed immature leaves.

Transcripts of *ZmIDD3 *and *ZmIDD7 *show nearly identical patterns; they are present in all tissues tested but highest amounts are seen in the stem region below the shoot apex (Figure 4B). In contrast, ZmIDD13 mRNA is abundant in flag leaf, tassel inflorescence and roots, but is barely detectable in stem, the shoot apex region or developing leaves (Figure 4B). Further, transcripts of all *ZmIDD *genes examined, except for *ID1*, are detected in the tassel inflorescence (Figure 4). Overall, the 7 maize *IDD *genes tested show wide-ranging expression in all shoot tissues tested and, unlike *ID1*, are not confined to immature leaf tissue.

### Isolation of putative *ID1 *orthologs from rice and sorghum

Before the first annotated draft of the rice genomic sequence was released, a genomic BAC (Bacterial Artificial Chromosome) library from the Clemson University Genomics Institute [[45]] with ten times genome coverage of the Japonica variety of *Oryza sativa *was screened for *IDD *genes. From this screen 137 BACs that hybridized to a full-length maize ID1 probe were identified. Of these, a subset of 14 BACs hybridized to a probe derived from *ID1 *sequences outside the ID domain (data not shown). A 6.6 kb fragment common to all 14 BACs was isolated and found to contain a coding region with a high level of similarity to maize *ID1*; this gene was designated *OsID*. Maize *ID1 *and rice *OsID *have a similar exon/intron structure common to most *IDD *genes; i.e., a small intron in the 5' region and a large intron that splits the fourth zinc finger (Figure 1). However, widespread regions of similarity were also found outside the ID-domain in both the N-terminal and C-terminal regions of the deduced OsID protein (Figure 2). That is, upstream of the putative nuclear localization sequence that delineates the left border of the ID domain and downstream of the fourth zinc finger (ZF4), which marks the right border.

The *OsID *gene was identified in the first draft of the rice genome [2,3] and no other gene with greater similarity to maize *ID1 *than *OsID *was detected. Although the current draft of the rice genome may not contain all genes [28], the fact that we only detected *OsID *in the BAC library screening suggests that it is the closest homolog to *ID1 *in the rice genome. Further, analysis of syntenic regions of the rice and maize genomes indicate that *OsID*, which is located on rice chromosome 10, lies in a region syntenic with maize chromosome 1, in the region where *ID1 *is located [28].

In addition to rice, we searched recently available *Sorghum bicolor *methylation filtered genomic sequences [29] and identified a closely related *IDD *gene, designated *SbID*, with significant homology to maize *ID1 *and rice *OsID*. As with OsID, SbID has extensive regions of identity outside the ID domain (Figure 3). Overall, the full-length SbID and OsID proteins are 84% and 61% identical to ID1 protein, respectively. Thus, the level of identity of sorghum SbID to maize ID1 is the highest of any gene identified so far. The level of similarity shared by *ID1*, *OsID *and *SbID*, indicate that these genes are closely related and could share homologous functions.

**Figure 5:**
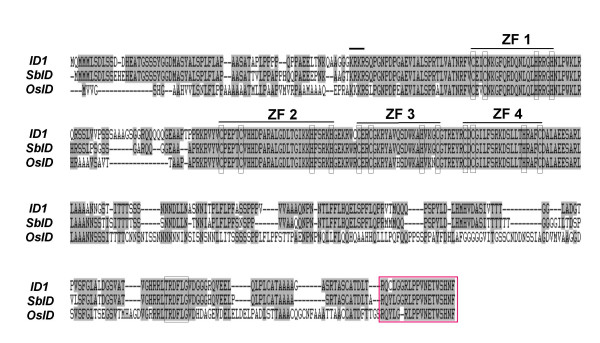
**Alignment of entire peptide sequences of ID1, OsID and SbID**. Amino acid residues shared by all three peptides are shaded. Each zinc finger is indicated with a light colored box; the putative NLS is shown by a dark bar and the TRDFLG motif is boxed. The boxed area with dotted line shows the C-terminal peptide region used to generate anti-ID1 specific antibody and corresponding sequences in rice and sorghum.

**Figure 6:**
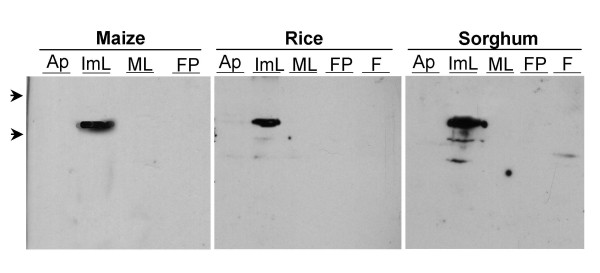
**Western blot analysis of ID1, OsID and SbID protein accumulation in maize, rice and sorghum tissues with anti-ID antibody**. Ap, apical region containing shoot meristem and leaf primordia; ImL, Immature leaves, approximately 2 to 8 cm above the apical region; ML, mature green leaf blades; FP, floral primordial tissues, which included ear tissue for maize, and yet to emerge spikelet primordia for rice and sorghum. Mature spikelet inflorescence, F, was also examined for rice and sorghum. Arrows at left indicate molecular weight standards of 84 kD and 61 kD.

To investigate whether these related grass genes have similar expression patterns, an anti-ID1 antibody specific for the C-terminal 20 amino acids of ID1 was used to analyse where OsID and SbID proteins are localized in developing rice and sorghum. The deduced C-terminal peptide sequences of SbID and OsID proteins are nearly identical to the C-terminal 20 amino acids of ID1; i.e., SbID and OsID share 19/20 and 18/20 amino acids, respectively (Figure 5). Figure 6 shows that this antibody cross-reacts with a single protein of the expected size in each species. Interestingly, similar to maize ID1, OsID and SbID proteins are detected exclusively in immature leaves and are absent in mature leaves and the region of the shoot apex (Figure 6). Furthermore, all three IDD proteins are absent in floral tissues in all species. Therefore the identical localization patterns of ID1 and its putative orthologs SbID and OsID show that all three genes have similar expression patterns.

### The Arabidopsis and rice genomes have similar numbers of *IDD *genes

The conserved ID-domain encoding sequence of maize *ID1 *was used to search for related genes in the Arabidopsis genome. We found 16 Arabidopsis ID-domain encoding genes with comparable levels of similarity to *ID1 *and the *IDD *genes from maize (Table 1). The Arabidopsis *IDD *genes, designated as *AtIDD*, share levels of amino acid identity with the ID-domain of maize *ID1 *that range from 63% to 84% (Table 1), but, as was found with the *ZmIDD *genes, there is little homology in sequences outside the 5' and 3' ID-domain coding regions, except in a few defined regions of certain genes, as described below (Figure 2). However, within this group of 16 genes, *AtIDD14*, *AtIDD15 *and *AtIDD16 *are more closely related to one another and diverge from the other 13 *AtIDD *genes, showing only 63 to 64% identity to *ID1 *(Table 1). Some of this divergence is the result of absence of conserved sequences outside regions encoding the ID domain. No other zinc finger families in Arabidopsis exhibit this level of similarity.

Englbrecht et al [20] defined a subfamily of 24 genes in Arabidopsis, called A1, that encode proteins with a configuration of four zinc fingers. They designated the 16 *AtIDD *genes defined here as a subclass of this group of 24, with the 13 genes most similar to maize *ID1 *termed A1a and 3 divergent members, *AtIDD14*, *AtIDD15 *and *AtIDD16*, comprising the A1b group. However, as a group the 16 *AtIDD *genes are much more divergent from the other 8 genes of the A1 grouping (A1c and A1d). That is, the zinc finger domains encoded by *AtIDD *genes are 63% to 84% identical to the maize ID1 zinc finger domain (Table 1), whereas they share less than 40% amino acid identity to the A1c and A1d members. Therefore we designate *AtIDD *genes as a separate family based on their similarity to maize *ID1*. Furthermore, the similarity between *AtIDD *genes extends beyond the zinc binding motifs and includes amino acid sequences that extend upstream and downstream of this region (Figure 3). Overall, the ID-domain is the defining feature shared by IDD proteins from Arabidopsis and maize.

Sequences searches of the current draft of the rice genome have revealed 15 *ID-*related genes, including *OsID*, which is comparable to the 16 *IDD *genes of Arabidopsis. This number of *ID1-related *genes in rice is in the range expected based on the 137 *IDD*-hybridizing BACs described above. Members of the rice family encoding ID-domain zinc finger proteins are designated *OsIDD *genes. The deduced proteins of *OsIDD *genes have a comparable level of similarity to each other and to IDD proteins of both maize and Arabidopsis (Table 1). In addition, *OsIDD *gene structures are similar to the structure of *ZmIDD *and *AtIDD *genes. For example, an intron splits the fourth zinc finger, ZF4, in all *IDD *genes from these three species, except for maize *ZmIDD3 *and *ZmIDD4 *genes (Figure 1). A common structural feature shared by all *IDD *genes is an intron that separates the 5' end of the gene from the ID-domain coding region (Figure 1).

A feature unique to the maize *ID1 *sequence is an intron that splits the second zinc finger, ZF2 (Figure 1). Even though *OsID *has extensive regions of identity to maize *ID1 *within the ID domain coding region as well as outside the domain, it does not have an intron within ZF2. The *SbID *gene, like *ID1*, is the only other *IDD *gene with an intron splitting the coding region of ZF2 (Figure 1). This shared feature of *ID1 *and *SbID *likely reflects the closer evolutionary distance between these two plants relative to rice; i.e., 16.5 million years between sorghum and maize vs. 70 million years between rice and maize [14]. Overall, comparison of the structure of maize and Arabidopsis *IDD *genes, including *ID1*, showed that, with a few exceptions, overall intron/exon structure is conserved (Figure 1).

### Other conserved motifs are found outside the ID domain of IDD proteins

Apart from the Iarge ID-domain, many IDD family members have small domains of similarity in the C-terminal region of the deduced protein (Figure 3). Two conserved regions that stand out are the "TRDFLG" domain, which is found in maize ID1 and many IDD proteins that are most similar to ID1, such as OsID and SbID, and the MSATALLQKAA domain. The TRDFLG motif is largely invariant, except in cases such as AtIDD1 and OsIDD1 where the sequence is TLDFLG, OsIDD7 and ZmIDD7, which contain the sequence TQDFLG, and the potato PCP1 deduced protein, which has the sequence TKDFLG (Figure 3).

The MSATALLQKAA motif is present in most of the Arabidopsis *IDD *genes and many maize and rice *IDD *genes, but it does not occur in ID1, OsID or SbID proteins (Figure 3). The presence of the TRDFLG sequence does not exclude the presence of MSTALLQKAA domains, and may reflect the modular nature of IDD regulatory proteins.

### Phylogenetic comparison of *IDD *genes from diverse plant species

**Figure 7:**
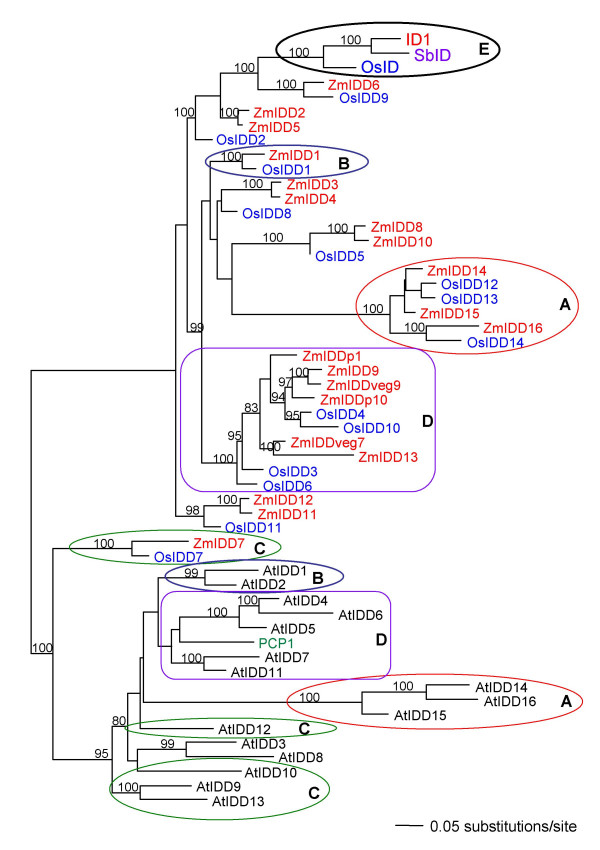
**Phylogenetic comparison of IDD genes from several plant species**. Mid-point rooted phylogram (branches proportional to the expected number of substitutions per site) based on genomic DNA sequences, reconstructed using Mr. Bayes. *IDD *genes found in maize (red), rice (blue) and Arabidopsis (black), as well as *PCP1 *from potato (*Solanum tuberosum*; green) and a potential ortholog of *ID1 *from *Sorghum bicolor*, *SbID *(purple), with Bayesian posterior probability values above 80% indicated above the branches.

Phylogenetic trees reconstructed from genomic DNA sequences from the conserved zinc finger region of all known *IDD *genes from maize, Arabidopsis and rice, along with the single *PCP1 *sequence from potato and SbID from sorghum showed extensive divergence of *ID1*-like sequences in all well-sampled species (Figure 7). No clear outgroup could be identified to root the tree in such a way that would allow conclusions about ancestral sequences, as the sequences are only alignable in the zinc finger domains that define the group and no other related sequences could be found that had a sufficient degree of overlap. However, the unrooted (not shown) and midpoint rooted phylograms both suggested independent diversification of the gene family in grasses compared to those in potato and Arabidopsis (Figure 7).

In Arabidopsis, *AtIDD14*, *AtIDD15*, and *AtIDD16 *(classified as A1b by Englbrecht et al [20]) were clearly divergent from the rest of the *AtIDD *sequences, whose relationships to one another (and to *PCP1*) were not as clearly resolved (as indicated by low Bayesian posterior probability values, pp). Similarly, within the grasses, *OsIDD12*, *OsIDD13*, *OsIDD14*, *ZmIDD-14*, *ZmIDD-15*, and *ZmIDD-16 *formed a strongly supported and distinctive grouping relative to the other sequences. Within the main sequence cluster, there were a number of well-supported groupings that included sequences from both rice and maize (1: *ZmIDD6 *with *OsIDD9*; 2: *ZmIDD1 *with *OsIDD1*; 3: *ZmIDD8 *and *ZmIDD10 *with *OsIDD5*; 4: *ZmIDDp1*, *ZmIDD9*, *ZmIDDveg9*, *ZmIDDp10*, *ZmIDDveg7 *and *ZmIDD13 *with *OsIDD4*, *OsIDD10*, *OsIDD3 *and *OsIDD6*; 5: *ZmIDD11 *and *ZmIDD12 *with *OsIDD11*; and 6: *ZmIDD7 *with *OsIDD7*), with *ID1*, *SbID *and *OsID *forming a strongly supported (pp = 100) and distinctive grouping most closely related to the *ZmIDD6*/*OsIDD9 *and then the *ZmIDD2/ZmIDD5 *sequence groups. Genomic sequences also identified *ZmIDD7 *and *OsIDD7 *as the most closely related to Arabidopsis *IDD *genes.

**Figure 8:**
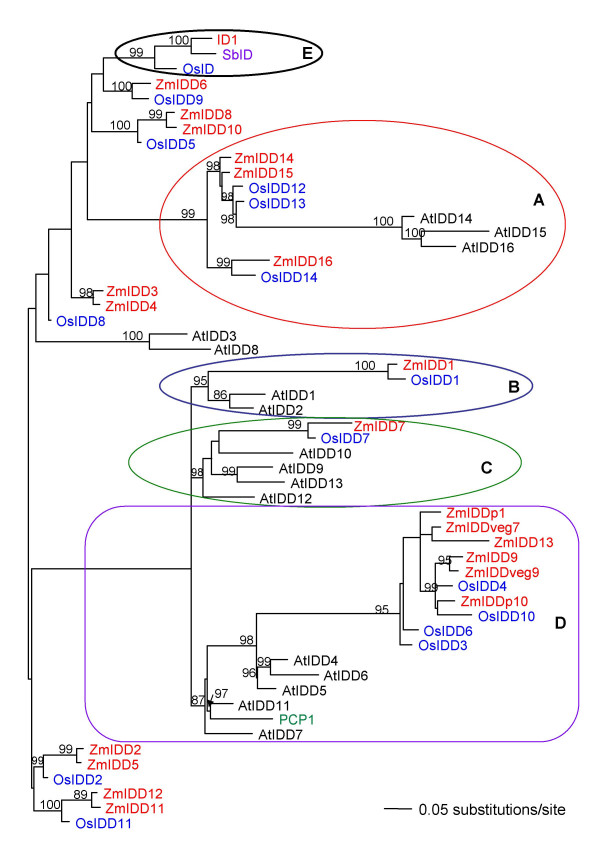
**Phylogenetic comparison of IDD amino acid sequences from several plant species**. Mid-point rooted phylogram based on amino acid sequences for the same region used in Figure 7. *IDD *genes found in maize (red), rice (blue) and Arabidopsis (black), as well as *PCP1 *from potato (*Solanum tuberosum*; green) and a potential ortholog of *ID1 *from *Sorghum bicolor*, *SbID *(purple), with Bayesian posterior probability values above 80% indicated above the branches. Circles of the same colour and labeled with the same letter on both trees highlight differences between the two sets of analyses; in particular, circled groups indicate amino acid groupings between Arabidopsis and grasses that were not apparent in genomic DNA analyses. Sequences enclosed by rectangles labeled 'D' form a less-well supported Arabidopsis-grass amino acid assemblage.

Phylogenetic analyses based on amino acid sequences gave different trees than those based on nucleotide sequences, but may reflect functional similarities among highly divergent proteins between species (compare Figures 7 and 8). In particular, the amino acid analyses indicated mixed clustering of genes between Arabidopsis and grasses whereas this was not seen in the genomic DNA analysis. Circled sets of sequences with the same letters are those that form highly supported "mixed" groups in the amino acid but not the nucleotide-based analyses. Note in particular that the two most divergent groups of sequences within each group clustered together in the amino acid analysis (circles labeled 'A'), based on sharing of unique domains not found in the other sequence types (although the branch lengths separating them are very long). Similarly, *AtIDD1 *and *AtIDD2 *appeared to be functionally related to *ZmIDD1 *and *OsIDD1 *(circles labeled 'B') and *ZmIDD7*/*OsIDD7 *appeared to be most functionally related to Arabidopsis sequences *AtIDD9*, *AtIDD10*, *AtIDD12 *and *AtIDD13 *(circles labeled 'C'). Sequences in rectangles labeled 'D' are quite divergent from one another but formed a weakly supported (pp = 87) grouping between grasses and Arabidopsis (and potato) sequences. Importantly, no functional equivalent was found for the *ID1*/*OsID*/*SbID *group (circle labeled 'E') in Arabidopsis and their relationship to the *ZmIDD6*/*OsIDD9 *and *ZmIDD8*/*ZmIDD10*/*OsIDD5 *groups was not well resolved in the amino acid analysis.

## Discussion

The maize *ID1 *flowering time gene encodes a putative transcription factor with four distinct types of zinc finger motifs [24]. Initial searches for similar genes revealed that *ID1 *is the first, genetically defined member of a highly conserved family of proteins that are found in all plant genome databases. The high level of similarity of *IDD *genes from species as diverse as monocots and dicots is a striking feature of this gene family. Here we have performed a comparative analysis of all available members of the *IDD *family from three species: maize, rice and Arabidopsis, as well as individual sequences from potato and sorghum. The complete genome sequences available for rice and Arabidopsis show that these species have 15 and 16 *IDD *genes, respectively. Analysis of GSS sequence data and an extensive maize cDNA/EST collection defined at least 21 *IDD *genes in maize, including *ID1*. The finding that maize has significantly more genes than either rice or Arabidopsis is likely due to the possible allotetraploid origin of the maize genome [14]. Given that the maize genome sequence is incomplete, it is possible that more *IDD *genes will be found. However, a recent evaluation of available maize genomic resources suggests that approximately 95% of the maize gene space has been at least partially uncovered [27]. A notable finding of our study is that, with the exception of one gene, unambiguous full-length coding regions were acquired for all *ZmIDD *genes, and the basic intron/exon structures were ascertained.

### The functions of most *IDD *genes are not known

The *IDD *genes most closely related to maize *ID1 *are the rice *OsID *gene and the sorghum *SbID *gene (Figure 5). *OsID *maps to chromosome 10 of the rice genome and does not coincide with any genes known to influence heading date, nor for other QTLs for flowering time in rice [30,31]. However, QTL analysis can be limited by the populations examined, therefore analysis of rice from different geographical locations may yet uncover QTLs that correspond to *OsID*. Transgenic experiments where *OsID *is over-expressed or its expression is eliminated or reduced by gene silencing techniques are in progress to reveal whether *OsID *has a role in controlling flowering time in rice, and also if maize *ID1 *can control flowering in a heterologous grass species (A. Kozaki and J. Colasanti, unpublished).

Apart from the role of *ID1 *in controlling the transition to flowering in maize, no other *IDD *gene with a genetically defined biological function has been reported. The *IDD *gene from potato, *PCP1*, was isolated in a screen for cDNAs that could rescue a yeast strain deficient in sucrose transport [32]. As a possible explanation for this finding the authors suggest that *PCP1 *may activate a previously unidentified sucrose transport system in yeast. *Saccharomyces cerevisiae *normally is not capable of this function because yeast excretes invertase to metabolize external disaccharides and converts them into hexoses that are transported into the cell. The *in planta *biological role of *PCP1 *remains unresolved, and no follow-up reports have been forthcoming. However, it is intriguing to speculate that *PCP1*, and perhaps other *IDD *genes, may have a role in sugar metabolism in higher plants. This is especially interesting in light of evidence that sucrose, perhaps in combination with other metabolites, may play an important role in signaling the transition to flowering [33]. If any of the *IDD *genes have a role in linking sucrose metabolism to plant development, it might be propitious to look for such a function amongst the maize, rice and Arabidopsis *IDD *genes that are most closely related to PCP1 (Figure 8, rectangle labeled 'D'). Of course such speculation needs to be supported by functional determination.

The extensive reverse genetic resources available for Arabidopsis allow for a functional genomic analysis of *AtIDD *genes. For example, we have recently discovered that loss of function of one of the divergent *IDD *genes, *AtIDD15*, has an effect on lateral shoot branching in Arabidopsis (H. Tanimoto, R. Tremblay, S. Chatfield and J. Colasanti, unpublished). Whether mutations in other *AtIDD *genes affect Arabidopsis growth and development is a subject of current investigation.

### Conserved regions exist outside the ID-domain

The designated ID-domain starts with a putative nuclear localization signal (usually KKKR or KRKR) and ends 9 amino acid residues beyond the last C residue of the fourth zinc finger (Figure 2). The high degree of conservation of this region in IDD proteins clearly delineates members of the *ID1*-related gene family. However, there are sequence motifs outside the ID-domain that allow for further sub-groupings of this gene family. A prominent conserved sequence found in maize ID1 and most other IDD genes is the TR/L/Q/DFLG sequence located near the C-terminus. In Arabidopsis, 13 of the 16 AtIDD proteins have either a TRDFLG or TLDFLG sequence, and 11 of the 15 rice OsIDD sequences have this motif. In both rice and Arabidopsis, IDD peptides without this sequence fall into a more divergent group, with the exception of OsIDD9 and ZmIDD6, which both lack this sequence and are closely related to each other (Figures 7 and 8). Clearly this motif has been conserved across lineages, suggesting a possible function such as in mediating protein-protein interactions. However, at present it is difficult to unambiguously assign activator or repressor function to domains within transcription factors without biochemical characterization [16]. BLAST analyses of short peptide sequences with TRDFLG, TLDFLG and TQDFLG as a query sequence show no other type of protein consistently harboring this motif except for *IDD *genes from higher plants.

The MSATALLQKAA sequence is another small motif outside of the ID-domain that is shared by some *ID1*-related gene family members. This motif is absent in maize ID1, yet is present in other ZmIDD peptides for which sequences are available. The divergent group of IDD proteins from maize, rice and Arabidopsis (the 'A' cluster in Figure 8) that do not have the TR/L/Q/DFLG motif similarly lack the MSATALLQKAA motif. It is interesting to note that these deduced proteins, which group together based on the similarities in their IDD sequences only, also show major differences outside the ID domain. This suggests that these sequences diverged early in angiosperm evolution and may have acquired separate and distinct functions.

### Maize *ID1 *has several distinctive features

Several lines of evidence suggest that maize *ID1 *is functionally distinct from *IDD *genes. First, maize *id1 *mutants have a dramatic late flowering phenotype, whereas mutations in other *ZmIDD *genes have not been reported. Second, a 45 amino acid spacer separates zinc fingers ZF1 and ZF2 of the ID1 protein, whereas the majority of IDD proteins examined here have 20 or 21 amino acids between the first two zinc fingers. The putative ID1 orthologs from rice (OsID) and sorghum (SbID) also have longer spacers between the first two zinc fingers than most IDD genes, with 29 and 35 amino acids, respectively. Other exceptions include *ZmIDD16 *and *OsIDD14*, but these genes belong to the divergent class of *IDD *genes found in all three species (the 'A' grouping in Figure 6) and therefore it is unlikely that they share a common function. Other exceptions include ZmIDD6 and OsIDD9, which have an additional 7 amino acids between ZF1 and ZF2. It is interesting to note that phylogenetic comparison reveals these two genes to be more closely related to each other than to other *IDD *genes from their own species and that *ZmIDD6 *and *OsIDD9 *appear to be the most closely related to the *ID1*/*SbID*/*OsID *cluster (Figures 7 and 8).

Finally, *ID1 *has a distinctive expression pattern that is not shared by the 7 *ZmIDD *genes examined here. So far, *ID1 *is the only maize *IDD *gene whose mRNA is detected exclusively in immature leaves, thus leading to speculation that *ID1 *regulates the transmission of leaf-derived floral inductive signals [10]. The finding that, similar to ID1, proteins encoded by the prospective *ID1 *orthologs from rice (*OsID*) and sorghum (*SbID*) are localized to immature leaves exclusively is intriguing because it suggests that these genes may regulate flowering time in related grass species. Genetic analysis of *OsID *and *SbID *is required to confirm this possibility.

One mechanism to target the activity of a regulatory protein is to restrict its expression to a particular tissue. A recent study found that one member of the 11 gene cytokinin oxidase family in rice, *OsCKX*2, had an expression pattern that differed from all other family members [34]. Although other members of the family had similar enzymatic activity in reducing cytokinin levels, the higher level of *OsCKX *expression in high yielding rice is suggested to explain phenotypic differences correlated with high yield rice. Similarly, the restriction of *ID1 *expression to developing leaves of maize may be explained by a specific function of this putative regulatory protein in controlling leaf derived floral inductive signals. No maize *IDD *genes examined here had an expression pattern similar to or restrictive as *ID1*. It is possible that one or more of the other *ZmIDD *genes that were not examined have expression patterns similar to *ID1*, however the degree of divergence of *ID1 *from all other *ZmIDD *genes suggest that they might not perform the same function, even if they had similar expression patterns. This can be tested once probes for all *ZmIDD *genes are available. Whether differences in the activity of each *IDD *gene is the result of its expression or because of subtle differences in regulatory action might be revealed by further genetic analyses of the function of each gene.

### Origin of the ID-domain gene family

Eukaryotic transcription factors can be classified into two basic groups based on their origins; those common to all lineages (plants, animals and fungi), and those specific to a particular lineage that evolved novel functions to carry out particular developmental needs of the organisms [35]. The IDD zinc finger proteins appear to be a hybrid of both types of transcription factor [24]. Zinc finger proteins are found in all eukaryotic lineages, suggesting that they are derived from a common eukaryote ancestor. However, the zinc finger family of transcription factors is diverse and can be divided into many subgroups based on a common structural feature; i.e., cysteine and/or histidine residues in a configuration that facilitates protein folding such that each finger interacts with a particular nucleotide sequence. Given the modular nature of zinc finger proteins, it is possible that during the course of evolution a "mixing and matching" of zinc finger units could occur to generate binding domains of a particular type. For example the CONSTANS family of transcription factors, some of which have an important role in photoperiod induced floral induction, appear to have a unique combination of zinc fingers [36].

We suggest that the arrangement of zinc finger modules in the ID domain has evolved to generate a class of zinc fingers that is unique to plants. That is, IDD proteins are derived from common zinc finger domains and act together to form a plant lineage-specific regulatory family. In addition to two canonical zinc fingers, IDD proteins also contain two unusual CCHC fingers, one of which is related to a zinc finger found in the *S. cerevisiae *SWI5 protein [24]. This combination of standard and unique zinc finger domains defines the IDD family found in all plants.

## Conclusion

Maize *ID1 *defines the *IDD *gene family that is present in higher plants. Analysis of the Arabidopsis and rice genomes reveals 16 and 15 *IDD *genes, respectively. Therefore the highly conserved *IDD *gene family is common to both monocots and dicots. From available genomic and EST/cDNA resources we have discovered 20 additional full-length *IDD *genes in maize, indicating that a large portion of the maize gene space may be represented. Within maize, expression of the *ID1 *gene is limited to a particular tissue, whereas expression patterns of several *ZmIDD *genes are broader. Phylogenetic comparison of *IDD *genes reveals that *ID1 *and putative *ID1 *orthologs from rice and sorghum form a subgroup distinct from other IDD genes. Further, the finding that OsID and SbID proteins, like ID1, are confined to immature leaf tissue during development supports the possibility that they have a similar function in regulating the floral transition in these species. Overall, it seems likely that the zinc finger configuration of IDD proteins was established in an ancestral plant species and has been maintained throughout evolution. The reason for the maintenance of the ID-domain suggests a general, plant-specific function but, apart from for the role of maize *ID1 *in regulating the transition to flowering, the functions of all other *IDD *genes remain to be elucidated.

## Methods

### Plant growth conditions

All plants (maize, rice and sorghum) were grown in soil pots in greenhouses from August to November with sodium vapor lamps providing supplemental lighting for 16 hours day/8 hours night. Temperature was maintained at 26°C during the day and 20°C at night.

### Isolation of maize *ID1 *and IDD cDNA clones

*ID1 *cDNA was isolated from a B73 immature leaf library made from 3 week old seedlings. Immature leaf tissue included all non-green leaves extending from 4 cm to 10 cm above the vegetative shoot apex. Total RNA was extracted from this tissue with TRIZOL reagent (Gibco-BRL) and polyA^+ ^mRNA was selected from total RNA with an Oligotex mRNA purification kit (Qiagen). A primary cDNA library of 1.3 × 10^6 ^plaques was constructed in the pBluescriptSK- vector using the λ-ZAP cDNA library construction kit (Stratagene). Approximately 10^6 ^plaques were plated on nylon filters and hybridized with a P^32^-labeled 666 bp probe amplified from the unique 3' end of the *ID1 *coding region as deduced from the genomic sequence. The cDNA clones from four positive plaques were isolated, analysed and found to correspond to the *ID1 *mRNA; three of the *ID1 *cDNA clones were full-length.

*ZmIDD *genes were isolated from similar screens as described above except that a 575 bp probe derived from the conserved region of the *ID1 *genomic sequence (from nucleotide 127 to 702) was used to screen ~10^6 ^plaques each from a vegetative shoot apex library (a gift from the Hake Laboratory, USDA-Plant Gene Expression Center, Albany, CA) and a 3-leaf seedling library (a gift of Alice Barkan, University of Oregon); both libraries were made with mRNA from maize inbred B73. A total of 23 clones were isolated and sequenced; 6 from the 3-leaf seedling library and 17 clones from the vegetative apex library. These represented 4 different *IDD *cDNAs, none of which corresponded to *ID1*. *ZmIDDp1 *and *ZmIDDp10 *cDNAs were found in both libraries, and *ZmIDDveg7 *and *ZmIDDveg9 *were found only in the vegetative apex library. Full-length sequences of each cDNA were determined and the sequences submitted to GenBank. Partial amino acid sequence of *ZmIDDp1 *was reported in the original paper describing isolation of the *ID1 *gene [22].

### Northern blotting and expression analysis of maize *ZmIDD *genes

Expression of *ID1 *and *ZmIDD *genes was examined by Northern analysis with poly A^+ ^selected mRNAs from various tissues as described previously [22]. Probes specific for *ZmIDDp1*, *ZmIDDp10*, *ZmIDDveg7 *and *ZmIDDveg9 *were derived from restriction fragments excised from each cDNA plasmids. The specific DNA fragments were SacI/XhoI (580 bp) from pZmIDDp1, SacI/XhoI (485 bp) from pZmIDDp10, SacI/XhoI (550 bp) from pZmIDDveg7 and XhoI (970 bp) from pZmIDDveg9. Probe DNA for other *ZmIDD *genes were isolated by PCR amplification with specific primers in non-conserved regions of each gene. Only 3 specific probes for the remaining 16 ZmIDD genes could be isolated from genomic DNA. The ZmIDD3 probe was amplified with 3L (AGGGACAGCTTCATCACG) and 3R (AATCCCTGGTCTCCTCCCTC), ZmIDD7 with 7L (CACACACGTCGGAAGAAGAATC) and 7R (TCCTCTGCTTCAGCTTCCATG) and ZmIDD13 with 13L (ACGTTAACCTCCTGGACATTG) and 13R (TACTAGTCTCCCGGACGCTG). Attempts to isolate PCR products for the remaining *ZmIDD *genes were not successful. A specific probe for the maize *CDC2 *gene (*ZmCDC2*) was derived from a cDNA clone as previously described [37]. The actin probe was amplified from B73 maize genomic DNA with primers described by Vollbrecht et al. [38]. All DNA fragments were purified from agarose gels and labeled with [^32^P]dCTP with a random primed labeling kit (Fermentas). Nylon membranes were stripped and probed sequentially with each probe for Northern blots in Figure 4A and 4B separately.

### Protein extraction and Western blot analysis

Proteins were extracted from vegetative tissues (mature leaf (ML), immature leaf (IL) and apical regions (Ap) of 3-week old maize (*Zea mays*) B73 seedlings, 6 to7 week old rice plants (*Oryza sativa*, var. Japonica) and 4 to 6 week red sorghum (*Sorghum bicolor*). Proteins from maize floral tissues were extracted from 2 cm ear primordia (FP). Proteins extracted from rice floral tissues included main inflorescence primordia (FP) at the 2 to 4 cm stage and mature floral spikelets before anther dehiscence (F); equivalent tissues were used for protein extraction from sorghum. Visualization of ID1, OsID and SbID proteins with anti-ID1 antibody by Western blotting required enrichment for nuclear proteins following a modification of the protocol by Steinmuller and Apel [39]. Briefly, plant tissues (0.5 to 1.0 gm) were collected, ground to a fine powder in liquid nitrogen and put in 5 mL of cold Isolation Buffer (IB: 20 mM Tris-HCl, ph 7.8, 250 mM sucrose, 5 mM MgCl_2_, 5 mM KCl, 40% (v/v) glycerol, 0.25% (v/v) TritonX-100, 0.1% (v/v) β-mercaptoethanol). This suspension was filtered through 50 μM nylon mesh, centrifuged at 6,000 g for 10 minutes and the pellet was washed twice with cold IB, and resuspended in 50 μL of IB. A portion of this was used for quantification with a protein assay kit (BioRad, Hercules, CA) and 50 μL of Laemmli loading buffer (62.5 mM Tris-HCl pH 6.8, 2% (w/v) SDS, 25% (v/v) glycerol, 0.01% (w/v) bromophenol blue) was added to the remainder. Duplicate samples of proteins (50 to 100 μg) were resolved on 11% (w/v) polyacrylamide gels and transferred to polyvinylidene fluoride (PVDF) membranes by semi-dry blotting (Owl Scientific), or stained with Coomassie blue to confirm protein quantity and assess protein integrity. For Western blots PVDF membranes were blocked with 5% (w/v) non-fat dry milk in Tris-buffered saline, TBS (20 mM Tris-buffered, 137 mM NaCl, pH 7.7) with 0.2% (v/v) Tween-20. Affinity-purified anti-ID1 antibody was used at 1/300 to 1/500 dilution in TBS-Tween/5% milk solution and incubated for 1 hour at room temperature. Blots were washed with TBS-Tween 6 times for 10 min each and then incubated with horseradish peroxidase-conjugated goat anti-rabbit antibody (Amersham) at 1/10,0000 dilution, and then washed as above. Bound antibodies were detected with Super Signal West Pico Chemiluminescent substrate (Pierce, Woburn, MA) and exposure to XAR X-ray film (Kodak).

### Genomic and protein sequence analysis alignment

TBLASTX searches were performed using databases available from the National Center for Biological Information web site. The nucleotide sequence of the ID domain of *ID1 *was used to search both *Arabidopsis thaliana *and *Oryza sativa *genomic databases for *IDD *genes. For maize *IDD *genes, the GSS database was used. Sequence reconstruction was performed using BioEdit Sequence Alignment Editor [40]. Alignments of deduced IDD proteins were performed using CLUSTALW software and manually corrected.

### Phylogenetic analysis of the *IDD *gene family

Nucleotide sequences were aligned by first translating to proteins and then using Se-Al version 2.0. Se-Al: Sequence alignment editor, version 1.0 alpha1 [46] was used to manually align protein motifs. Most sequences were alignable only in the region of the ID domain. Sequences were pruned to include only these shared domains, which included sequence from the NLS motif at the amino terminus to 9 amino acids after the last C residue of ZF4 (and any intervening regions that could be aligned for all sequences without ambiguity), resulting in a 708 bp fragment after back-translating to nucleotide sequences. Phylogenetic trees were reconstructed for both nucleotide and amino acid alignments using Mr. Bayes version 3.0 [41]. For nucleotide sequences, the most appropriate model of evolution to explain the data (general time reversible model estimating separate rates of change for each type of nucleotide substitution, with both invariant sites and gamma distribution shape parameter estimated to account for rate heterogeneity) was determined using Model Test version 3.5 [42], in conjunction with PAUP* 4.0b10 [42], PAUP*4.0b10. Sinauer Associates Inc., Massachusetts). For amino acid sequences, the mixed model available in Mr. Bayes, which assigns equal prior probability to 10 different models of amino acid substitution, was used as the underlying model of evolution. Analyses were run with the following parameters: number of generations = 1,000,000; sampling frequency = 10 generations; number of chains = 4. Posterior probability values (which allow assessment of relative support for particular branching relationships) were computed after discarding the first 1500 generations (burn-in period) and computing a consensus of trees saved in the remaining generations. Trees were visualized using PAUP*, with branch lengths optimized using maximum likelihood.

## Authors' contributions

JC performed initial sequence analysis, isolated cDNAs from maize libraries, isolated rice sequences from BAC libraries, performed initial Northern analysis, and drafted the manuscript, RT extracted and organized all genomic sequences and performed manual sequence alignments, AYMW performed and analysed Western blotting, VC isolated maize cDNA sequences and performed Northern blots, AK analysed rice sequences and performed initial phylogenetic and synteny analysis, BKM performed phylogenetic analysis and interpretation.

## Figures and Tables

**Table 1:** The *IDD *gene family. Deduced amino acid size, degree of identity to ID1 and accession numbers for *IDD *genes from maize, rice and Arabidopsis,* PCP1 *from potato and *SbID *from sorghum. (^a^) represents % identity/similarity to all ID domains relative to ID1 without the 23 amino acid spacer. (A) refers to the phylogenetic grouping shown in Figures 7 and 8.

**IDD gene**	**# amino acids**	**% identity to ID1**^a^	**Accession #**
**ID1**	436	100	AF058757
ZmIDDp1	542	81/93	AY754867
*ZmIDDp10*	583	81/92	AY754864
*ZmIDDveg7*	518	81/92	AY754865
*ZmIDDveg9*	588	82/92	AY754866
*ZmIDD1*	539	85/94	N/A
ZmIDD2	497	87/94	N/A
*ZmIDD3*	398	85/92	N/A
*ZmIDD4*	437	85/92	N/A
*ZmIDD5*	467	85/92	N/A
*ZmIDD6*	475	83/92	N/A
*ZmIDD7*	510	76/91	N/A
*ZmIDD8*	424*	81/88	N/A
*ZmIDD9*	600	82/92	N/A
*ZmIDD10*	343	80/86	N/A
*ZmIDD11*	480	82/91	N/A
*ZmIDD12*	468	82/91	N/A
*ZmIDD13*	554	75/88	N/A
*ZmIDD14 *(A)	498	60/77	N/A
*ZmIDD15 *(A)	518	61/77	N/A
*ZmIDD16 *(A)	458	63/79	N/A
			
AtIDD1	500	83/93	At5g66730
AtIDD2	452	81/93	At3g50700
AtIDD3	506	84/92	At1g03840
AtIDD4	516	77/94	At2g02080
AtIDD5	602	78/93	At2g02070
AtIDD6	467	78/94	At1g14580
AtIDD7	455	83/94	At1g55110
AtIDD8	466	82/91	At5g44160
AtIDD9	446	82/92	At3g45260
AtIDD10	503	82/91	At5g03150
AtIDD11	513	82/94	At3g13810
AtIDD12	402	82/91	At4g02670
AtIDD13	455	76/92	At5g60470
AtIDD14 (A)	419	64/78	At1g68130
AtIDD15 (A)	439	64/77	At2g01940
AtIDD16 (A)	385	63/76	At1g25250
			
**OsID**	475	94/96	Os10g28330
OsIDD1	552	85/94	Os03g10140
OsIDD2	487	86/92	Os01g09850
OsIDD3	521	82/93	Os09g38340
OsIDD4	615	82/92	Os02g45050
OsIDD5	633	81/89	Os07g39310
OsIDD6	550	81/93	Os08g44050
OsIDD7	492	77/91	Os02g31890
OsIDD8	495	85/92	Os01g14010
OsIDD9	476	82/91	Os01g70870
OsIDD10	551	8292	Os04g47860
OsIDD11	535	80/90	Os01g39110
OsIDD12 (A)	586	63/79	Os08g36390
OsIDD13 (A)	504	63/79	Os09g27650
OsIDD14 (A)	459	63/79	Os03g13400
			
**SbID**	428	99/99	N/A
PCP1 (potato)	509	81/92	X82328
